# S10-1 Developing and monitoring physical activity policy: 1990 to today

**DOI:** 10.1093/eurpub/ckac093.050

**Published:** 2022-08-29

**Authors:** Michael Pratt

**Affiliations:** Institute for Public Health and MPH Program Department of Family Medicine and Public Health, University of California San Diego , San Diego, USA

**Keywords:** Policy research, global, national policy, implementation

## Abstract

**Background:**

Physical inactivity accounts for as many as 5 million deaths per year globally but has yet to be addressed effectively by most governments or the World Health Organization (WHO).

**Description of the problem:**

Reasonable evidence for effective strategies exist, and several countries have implemented consistent public health policies and programs that have increased population prevalence of regular physical activity. In 2018 WHO launched a Global Action Plan for Physical Activity (GAPPA). However, most countries do not have effective public health policies in place for physical activity. Evaluating the history of national and global policies to understand how some countries have developed sound public health programs for physical activity while most have not requires monitoring and evaluating policy, programmatic, and perhaps even research indicators for physical activity. Only recently have there been systematic efforts to do so.

**Results:**

Several key questions about physical activity policy, starting with an examination of the evolution of public health physical activity policy and policy research from when it was first explicitly identified in the 1990s to the present will be addressed. Key recommendations, global statements, research reviews and action plans will be summarized to provide a clear picture of the evolution of this important aspect of public health. Evidence on the implementation of policy at national and global levels will also be reviewed. We will examine whether it feasible to track physical activity policy at the global and country levels, if the existence of ?good? physical activity policy is associated with less physical inactivity, how policy indicators may be used for advocacy and guidance.

**Lessons:**

Physical activity policy for public health is a relatively new concept that has evolved significantly in the last three decades with formal national and global policy statements, research, and evaluation efforts all increasing markedly since 1990.

**Main Message:**

Despite notable growth in policy research and the development of sophisticated national and global policies and action plans, implementation of effective national and global policies and programs for physical activity remains limited.

